# Carbapenemase-producing Organism in Food, 2014

**DOI:** 10.3201/eid2007.140534

**Published:** 2014-07

**Authors:** Joseph E. Rubin, Samantha Ekanayake, Champika Fernando

**Affiliations:** University of Saskatchewan, Saskatoon, Saskatchewan, Canada

**Keywords:** carbapenemase, antimicrobial drug resistance, VIM-2, Pseudomonas, foodborne

**To the Editor:** Carbapenem antimicrobial drugs are the line of defense against multidrug-resistant gram-negative bacterial infections. The global emergence of carbapenemase-producing organisms is a public health emergency because these enzymes confer resistance to nearly all β-lactam drugs and are often associated with multidrug or pandrug resistance (*1*). Alarmingly, reports of carbapenemase-producing organisms from environmental and animal sources, including food animals, are increasing (*1*). Recently, clinical isolates of *Salmonella enterica* serotype Kentucky that produce VIM-2 and OXA-48 were reportedly isolated from patients in France with a travel history to Africa and the Middle East, suggesting foodborne transmission of carbapenemase producers (*2*).

To the best of our knowledge, before this report no foodborne carbapenemase-producing organisms had been identified in Canada and the United States, although the scope of antimicrobial drug resistance surveillance programs is limited to major agricultural products (poultry, beef, and pork) (*3*,*4*). In our modern, ethnically diverse societies, niche-market meat products, including imported foods, are becoming increasingly common. Worldwide dissemination of the *Klebsiella pneumoniae*, VIM, OXA, and New Delhi metallo-β-lactamase type carbapenemases among humans has been facilitated by intercontinental passenger travel, but the role of the global food trade in this dissemination has not been investigated (*5*,*6*). We describe a carbapenemase-producing organism isolated from a squid purchased from the seafood section of a food store.

Among other items, the squid was purchased from a Chinese grocery store in Saskatoon, Canada, in January 2014 as part of a drug-resistance surveillance pilot study. Although no country-of-origin labeling was available for inspection, the store owner reported that, according to the distributor, this squid originated in South Korea. An organism with 95.5% sequence identity to *Pseudomonas fluorescens* was isolated on Mueller-Hinton agar with 2 μg/mL meropenem and identified by partial sequencing of the *cpn*60 gene (GenBank accession no. KJ606641). Although the organism was not extensively resistant, it was resistant to all β-lactam drugs tested including ertapenem (Table). PCR amplification and sequencing confirmed that this organism contained VIM-2 carbapenemase (GenBank accession no. KJ625238).

**Table:** Antimicrobial drug susceptibility of a VIM-2 producing *Pseudomonas fluorescens*–like organism isolated from food (squid), Saskatoon, Canada, January 2014

**Antimicrobial drug**	**MIC**
**Ampicilin**	>32
**Amoxicillin + clavulanic acid**	>32
**Cefoxitin**	>32
**Ceftiofur**	>8
**Ceftriaxone**	>64
**Azithromycin**	16
**Chloramphenicol**	16
**Tetracycline**	≤4
**Naladixic acid**	16
**Ciprofloxacin**	0.06
**Gentamicin**	≤0.25
**Kanamycin**	16
**Streptomycin**	≤32
**Sulfisoxazole**	32
**Trimethoprim + sulfamethoxazole**	0.5
Ertapenem*	>32
Tigecycline*	0.125
Colistin*	3

*MICs determined by Etest; all others were determined by broth microdilution.

The presence of carbapenemase-producing organisms in the food supply is alarming. Although this organism may not be a pathogen, its contribution to the resistome and the potential for lateral gene transfer to clinically relevant bacteria is certainly a cause for concern. This finding indicates that the risk for exposure to carbapenemases extends beyond persons with particular travel histories, previous antimicrobial drug use, or hospitalization and into the general public. There is an urgent need for expanded resistance surveillance for carbapenemase-producing organisms and their resistance plasmids in food products that are not captured under current programs.

## References

[1] Woodford N, Wareham DW, Guerra B, Teale C. Carbapenemase-producing *Enterobacteriaceae* and non-*Enterobacteriaceae* from animals and the environment: an emerging public health risk of our own making? J Antimicrob Chemother. 2014;69:287–91. 10.1093/jac/dkt392 24092657

[2] Le Hello S, Harrois D, Bouchrif B, Sontag L, Elhani D, Guibert V, Highly drug-resistant *Salmonella enterica* serotype Kentucky ST198–X1: a microbiological study. Lancet Infect Dis. 2013;13:672–9. 10.1016/S1473-3099(13)70124-5 23721756

[3] Canada Go. Canadian Integrated Program for Antimicrobial Resistance Surveillance (CIPARS): antimicrobial resistance short report; 2011 [cited 2014 Apr 23]. http://publications.gc.ca/collections/collection_2013/aspc-phac/HP2-4-2-2011-eng.pdf

[4] US Food and Drug Administration. Retail meat report: National Antimicrobial Resistance Monitoring System; 2011 [cited 2014 Apr 23]. http://www.fda.gov/downloads/AnimalVeterinary/SafetyHealth/AntimicrobialResistance/NationalAntimicrobialResistanceMonitoringSystem/UCM334834.pdf

[5] van der Bij AK, Pitout JD. The role of international travel in the worldwide spread of multiresistant *Enterobacteriaceae.* J Antimicrob Chemother. 2012;67:2090–100. 10.1093/jac/dks214 22678728

[6] Nordmann P, Naas T, Poirel L. Global spread of carbapenemase-producing *Enterobacteriaceae.* Emerg Infect Dis. 2011;17:1791–8. 10.3201/eid1710.110655 22000347 PMC3310682

